# Michigan cohorts to determine associations of maternal pre-pregnancy body mass index with pregnancy and infant gastrointestinal microbial communities: Late pregnancy and early infancy

**DOI:** 10.1371/journal.pone.0213733

**Published:** 2019-03-18

**Authors:** Kameron Y. Sugino, Nigel Paneth, Sarah S. Comstock

**Affiliations:** 1 Department of Food Science and Human Nutrition, Michigan State University, East Lansing, MI, United States of America; 2 Department of Epidemiology and Biostatistics, College of Human Medicine, Michigan State University, East Lansing, MI, United States of America; 3 Department of Pediatrics and Human Development, College of Human Medicine, Michigan State University, East Lansing, MI, United States of America; University of Missouri Columbia, UNITED STATES

## Abstract

**Background:**

About 25% of women in the United States are obese prior to becoming pregnant. Although there is some knowledge about the relationship between the gastrointestinal microbiota and obesity, little is known about the relationship between pre-pregnancy obesity and the gastrointestinal microbiota in pregnancy or its impact on infant gut microbiota. However, the composition of the gut microbiota early in life may influence childhood health. Thus, the objective of this research was to identify associations between maternal pre-pregnancy obesity and the pregnancy (n = 39) or early infancy (n = 39) microbiotas.

**Results:**

Fecal bacterial communities from overweight women had lower microbiota diversity (Chao1: p = 0.02; inverse Simpson: p = 0.05; Shannon: p = 0.02) than communities from normal weight or obese women. The within-group microbiota composition of overweight women differed from those of normal and obese women at the genus and phylum levels (p = 0.003 and p = 0.02, respectively). Pre-pregnancy overweight women had higher abundances of *Bacteroides* and lower *Phascolarctobacterium* than women who were normal weight or obese prior to becoming pregnant. Normal weight women had lower abundances of *Acidaminococcus* and *Dialister* than overweight and obese women. Infant community composition tended to differ in membership (Sorensen index) by maternal pre-pregnancy BMI category, and significantly differed by delivery mode and breastfeeding exclusivity (p = 0.06, p = 0.001, p = 0.008, respectively). Infants from normal weight women had lower abundances of *Megasphaera* than infants from overweight or obese women. *Streptococcus* was lowest in infants from overweight women, and *Staphylococcus* was lowest in infants from obese women.

**Conclusion:**

Maternal and infant microbiotas are associated with and might be affected by maternal pre-pregnancy BMI. Future work should determine if there are also functional differences in the infant microbiome, if those functional differences are related to maternal pre-pregnancy BMI, and whether differences in composition or traits persist over time.

## Introduction

The gut hosts a diverse community of microbes that interact with biological functions in both humans and animals. For example, the gut microbiota impacts immune system development, digestion of food components and influences weight gain [1–4]. In mice, the presence of a gut microbiota promotes increased adiposity and weight gain, possibly by increasing the amount of energy extracted and absorbed from food [1, 3, 5, 6]. Similarly, germ-free mice transplanted with the microbiota from an obese human twin have more body fat compared to mice that receive a microbiota transplant from the lean twin [1]. In healthy human adults, there is evidence that shifts in the Firmicutes and Bacteroidetes abundances are associated with weight gain and obesity, though these shifts in phyla abundances are not consistent between studies [1, 7, 8]. Thus, although there is strong evidence for a relationship between gut microbiota and obesity in mice, the evidence in humans is weak [8]. To address this question more definitively in humans, analysis of these communities during pregnancy and early infancy [9, 10] can inform how the gut microbiota is associated with maternal pre-pregnancy BMI. While it is known that maternal pre-pregnancy BMI is associated with infant development and child weight [11, 12], it’s unknown why these relationships exist. However, these effects may be mediated through the pregnancy and infancy microbiotas [13].

In the United States, 50% of women giving birth in 2014 were overweight or obese [14]. In adults, there is an association between higher BMI and differences in the microbiome, such as low alpha diversity, altered community structure and changes in the functional capacity of the metagenome [15]. Similar changes have been observed over the course of pregnancy [10] possibly due to alterations in hormone levels, the immune system and metabolism [16]. In the first trimester, the gut microbiota displays a significantly lower alpha and higher beta diversity concurrent with higher abundances of the phyla Proteobacteria and Actinobacteria compared to the third trimester [17]. There is evidence of differences in the microbiome during pregnancy based on body weight [18, 19] and gestational weight gain [20], but the evidence on pre-pregnancy weight and microbiota in late pregnancy is contradictory. Some studies found no association between pre-pregnancy BMI and the pregnancy microbiota [10, 20], while another found overweight/obese women had a lower abundance of *Parabacteroides* and *Bifidobacterium* compared to normal weight [21]. This suggests that pregnancy may be an important driving factor of the maternal gut microbiota in some populations, which can affect the bacteria infants are exposed to during and after birth [22].

The infant microbiota increases in bacterial abundance and becomes more diverse over time as the infant is exposed to changes in diet and environment [23, 24]. One of the earliest environmental exposures is mode of delivery. In the first week of life, vaginally born infants have a higher proportion of their communities in common with their mothers’ intestinal communities such as *Bifidobacterium*, *Parabacteroides* and *Escherichia/Shigella* compared to infants born via cesarean delivery [4]. In contrast, the microbiota of infants born via C-section are enriched in skin, oral and environmental bacteria such as *Enterobacter*, *Staphylococcus* and *Streptococcus* [4, 25, 26]. At 4 and 12 months, the association between mode of delivery and gut microbiota is still present, but the differences decrease over time [4]. Breastfeeding also affects gut microbiota [27]. *Lactobacilli* and *Bifidobacterium*, both of which utilize human milk oligosaccharides tend to dominate the gut microbiota of breastfed infants [4, 28, 29].

High maternal pre-pregnancy BMI has been linked to an increased risk of high childhood BMI [30]. Obese children are more likely to be obese adults with chronic medical problems [31]. The infant microbiome may explain predisposition to weight gain and metabolic dysregulation and may also explain the effects that breastfeeding [32] and cesarean delivery [33] have on obesity risk during childhood. A study by Tun et al. found that children at 1 year of age were 3 times more likely to be overweight if they were born vaginally from pre-pregnancy overweight or obese mothers, which was associated with an increased abundance of *Lachnospiraceae* [34]. Other studies that have assessed maternal pre-pregnancy BMI and the infant microbiota found that beta diversity was significantly different between infants from normal and overweight/obese groups [21] and another found that only vaginally born neonates had altered microbiota based on pre-pregnancy BMI [35].

Here we use samples from women in their third trimester of pregnancy and their infants to investigate associations between maternal pre-pregnancy BMI and the pregnancy or infant microbiotas, controlling for effects of other factors such as breastfeeding and delivery mode.

## Materials and methods

### Subjects

Women were enrolled as part of the ARCH_GUT_ or BABY_GUT_ cohorts. ARCH_GUT_ is part of a larger study (ARCH, the Archive for Research in Child Health) in Lansing and Traverse City, MI. The ARCH cohort recruited participants from one clinic in each location and BABY_GUT_ recruited participants from several clinics in Lansing. All ARCH_GUT_ and BABY_GUT_ participants provided written informed consent upon enrollment. The Michigan State University Human Research Protection Program approved the studies (IRB 15–1240 and 14-170M).

### Sample collection

Fecal samples were collected by the women (n = 42) at their homes during the third trimester; mothers also collected fecal samples from their infant at home when the infant was around 1 week of age (n = 43). Of these, only paired samples (dyads with both a pregnancy and an infancy sample) were included in the analysis. Furthermore, for a single pregnancy sample with two infancy samples (twin pair), only the pregnancy and first-born twin samples were included in the final analyses. The final sample size was n = 39 dyads. Infants were a median of 8.5 days old at the time of sample collection. Collection kits were assembled at the research facility and sent to the participants via mail. The collection kits included instructions for taking a fecal sample, a ParaPak tube for sample collection (Meridian Bioscience, 900312), a box with postage to return the sample, a commode collection kit for the third trimester samples (Thermo Fisher, 02-544-208) or diapers for the infant sample. Samples were sent to the lab by mail or retrieved from the home, and fecal aliquots were stored at -80°C upon reaching the lab. The average time from sample collection to receipt was 4.2±2.2 days (median of 4 days) for the pregnancy samples and 5.1±3.3 days (median of 4 days) for the infant samples. There were no significant differences in shipping time across BMI categories for either pregnancy or infant samples (Table 1). Participants completed questionnaires at the time of enrollment and at the time of each sample collection.

**Table 1:** Participant characteristics.

Pregnant Women	All	18.5 ≤ BMI < 25	25 ≤ BMI < 30	BMI ≥ 30	p-value
Participants, n	39	12	11	16	
Pre-pregnancy BMI (kg m^-2)^1^	28.5 ± 5.5	22.7 ± 1.4^c^	26.7 ± 1.5^b^	34.1 ± 3.3^a^	<0.001
Maternal Age (years)^1^	31.4 ± 4.5	33.3 ± 2.5	29.6 ± 5.1^4^	31.0 ± 5.1	0.15
Currently on Antibiotics^3^	1 (2.6)	1 (8.3)	0 (0.0)	0 (0.0)	0.32
Parity^2^	2.0 (1–6)	2.0 (1–5)	2.0 (1–3)	2.0 (1–6)	0.96
Sample Shipping Time (days)^2^	4.0 (0–11)	4.0 (1–7)	4.0 (0–11)	4.5 (2–7)	0.96
**Infants**	All	18.5 ≤ BMI < 25	25 ≤ BMI < 30	BMI ≥ 30	p-value
Vaginal Delivery^3^	26 (66.7)	11 (91.7)	5 (45.5)	10 (62.5)	0.06
Female^3^	14 (35.9)	3 (25.0)	4 (36.4)	7 (43.8)	0.59
Exclusively Breastfed^3^	24 (61.5)	11 (91.7)^a^	6 (54.5)^ab^	7 (43.8)^b^	0.03
Antibiotic Exposure Since Birth^3^^3^	4 (10.3)	0 (0.0)	2 (18.2)	2 (12.5)	0.33
Infant Sample Shipping Time (days)^2^	4.0 (1–15)	3.0 (2–11)^4^	5.0 (1–15)	5.0 (2–14)^4^	0.85
Infant Age at Sampling Time (days)^2^	8.5 (2–111)	5.5 (2–57)	10.0 (7–60)	17.0 (3–111)^4^	0.05

Values in a row that do not contain the same superscript are significantly different, p<0.05

^1^mean ± SD

^2^median (range)

^3^n (%)

^4^Missing information for one sample

### DNA extraction and amplification

DNA extractions were performed using the MoBio Powersoil DNA Isolation kit (Qiagen MoBio, Carlsbad, CA) per the Human Microbiome Project’s protocol [36] with a few alterations: after incubating in C3 solution, the samples were centrifuged for 2 minutes and the DNA was eluted from the spin columns with 50 μL of low EDTA TE buffer (IDT, Coralville, IA) heated to 55°C.

Barcoded primers were used to amplify the V4 region of the 16S rRNA gene following the mothur wet lab documentation [37]. Primers SB501-SB508 and SA701-SA712 were ordered from IDT (Coralville, IA). PCR amplification also followed the wet lab protocol outlined in the mothur documentation [37]. Briefly, Accuprime Pfx Supermix (ThermoFisher, Waltham, MA) was mixed with up to 10 ng of template DNA and the primer pair (final concentration of 500 nM for both the forward and reverse primers) in a final reaction volume of 20 μL. Reactions were performed in triplicate and amplified using the following thermocycler settings: (1x) 2 min at 95°C; (30x) 20 s at 95°C, 15 s at 55°C, 5 min at 72°C; 10 min 72°C. Amplification success was checked by electrophoresis on a 1% agarose gel run at 200 V for 30 min. Successful amplification triplicates were pooled and purified using Agencourt AMPure XP (Beckman Coulter, Brea, CA) with the following alterations to the protocol: 0.7 times the sample volume of AMPure was used for purification and 16S rRNA DNA was eluted using 25 μL of low EDTA TE buffer (IDT, Coralville, IA). This lower volume of AMPure excludes fragments of DNA with a lower base count than the amplified DNA. After purification, the concentration of 16S rRNA gene amplicons was quantified using the Quant-IT dsDNA assay kit (Invitrogen, Carlsbad, CA). Purified 16S rRNA amplicons were pooled and quality checked using an Agilent 2100 Bioanalyzer with the High Sensitivity DNA Chip (Agilent, 5067–4626). For sequencing, equal amounts (in nanograms) of the purified 16S samples were pooled. The pooled DNA was diluted to 2.5 ng/μL and submitted to the Michigan State University Research Technology Support Facility Genomics Core for paired-end 250 base-pair sequencing on the Illumina MiSeq platform using V2 chemistry. Sequences have been deposited in the NCBI SRA under accession number PRJNA506270.

### Processing and analysis of sequence data

Sequence reads were processed in mothur using the Illumina MiSeq SOP [37] and operational taxonomic unit (OTU) taxonomies were assigned by phylotype using the SILVA reference taxonomy (release 128) [38]. Read processing was done in mothur using the High-Performance Computing Cluster at Michigan State University. Sample reads were rarefied to 15000 reads per sample before further analysis and rarefaction curves confirmed adequate community coverage.

### Data analysis

The pregnant women were classified by pre-pregnancy BMI categories of normal (18.5≤BMI<25), overweight (25≤BMI<30) or obese (BMI≥30). Pre-pregnancy BMI was calculated from the self-reported height and weight of the participants [39]. Breast milk in the diet was reported by mothers who completed a published survey that separates the infant diet into seven levels of human milk exposure (100%, 80%, 50–80%, 50%, 20–50%, 20% and 0%) [40]. However, because of insufficient sample size for each of the groups, diet was categorized as exclusively or non-exclusively receiving human milk in the diet, either from the breast or a bottle. Infants whose mothers reported the infant had been given any antibiotic since birth were considered antibiotic exposed (n = 4, 2 in overweight and 2 in obese, Table 1). However, none of these infants were taking antibiotics at the time the sample was collected. Alpha (within-sample) diversity (Chao1, inverse Simpson and Shannon) was calculated in R [41] with the vegan package [42] and compared using a Kruskal-Wallis test or a Spearman correlation test. Pairwise tests between BMI categories was performed using a Dunn test. Sorensen (community composition) and Bray-Curtis (community structure) dissimilarities were calculated in R from the abundance data using the vegan package and ordinated using principle coordinate analysis (PCoA). Permutational multivariate analysis of variance (PERMANOVA) was performed using the adonis function to test for significant differences in beta-diversity. The adonis2 function was used to adjust for covariates in the pregnant women and infant data. For the women, maternal age, cohort (ARCH_GUT_ or BABY_GUT_) and shipping time were included as covariates with pre-pregnancy BMI. For the infants, shipping time, breastfeeding, sex, cohort, infant age and delivery mode were covariates with maternal pre-pregnancy BMI. Permutation of dispersion (PERMDISP—betadisper function in the vegan package) was used to test for differences in sample dispersion. Individual taxa were compared across BMI categories using a negative binomial model in the MASS package [43] on the taxa that composed >1% abundance on average. The Benjamini-Hochberg method was used for false discovery rate correction. P-values less than 0.05 were considered significant, and p-values less than 0.10 were considered a trend.

## Results

### Subject characteristics

We collected fecal samples from 39 dyads. Of these, there was one twin birth, and only one of the twins was included in this analysis. In total, there were 12 normal weight, 11 overweight and 16 obese women. Among the women, none of the self-reported characteristics differed by BMI category (Table 1). Fewer infants of obese women consumed breastmilk exclusively (Table 1). A higher percent of infants born to normal weight mothers tended to be born vaginally compared to infants of overweight and obese mothers (Table 1). Infant age at the time of sampling ranged from 2 to 111 days (median = 8.5 days). Infants born to normal weight women tended to be younger at the time of sampling than those born to overweight or obese women (Table 1). The characteristics of participants in ARCH_GUT_ (n = 24) and BABY_GUT_ (n = 15) were similar (Table 2).

**Table 2:** Cohort characteristics.

**Pregnant Women**	ARCH	BABY	p-value
N	24	15	
Pre-pregnancy BMI (kg m^-2)^**1**^	29.0 ± 5.4	27.7 ± 5.8	0.47
Normal^**3**^	7 (29.2)	5 (33.3)	0.29
Overweight^**3**^	5 (20.8)	6 (40.0)	
Obese^**3**^	12 (50.0)	4 (26.7)	
Maternal Age (years)^**1**^	31.3 ± 3.8	31.4 ± 5.7^4^	0.95
Currently on Antibiotics^**3**^	0 (0.0)	1 (6.7)	0.81
Parity^**2**^	2.0 (1–6)	2.0 (1–3)	0.99
Sample Shipping Time (days)^**2**^	4.0 (1–7)	5.0 (0–11)	0.09
**Infants**	ARCH	BABY	p-value
Vaginal Delivery^**3**^	17 (70.8)	9 (60.0)	0.73
Female^**3**^	11 (45.8)	3 (20.0)	0.2
Exclusively Breastfed^**3**^	14 (58.3)	10 (66.7)	0.86
Antibiotic Exposure Since Birth^**3**^	1 (4.2)	3 (20.0)	0.3
Infant Sample Shipping Time (days)^**2**^	4.0 (2–14)^4^	6.0 (1–15)^4^	0.2
Infant Age at Sampling Time (days)^**2**^	20.7 ± 26.2^4^	13.9 ± 14.4	0.66

^1^mean ± SD

^2^median (range)

^3^n (%)

^4^Missing information for one sample

### Alpha diversity

Pregnant women had significantly higher microbiota diversity at the genus level than their infants, as measured by either Chao1 (p<0.001), inverse Simpson (p<0.001) and Shannon indices (p<0.001). The fecal bacterial communities of women who were overweight prior to becoming pregnant were less rich (Chao1), more even (inverse Simpson) and had a lower diversity (Shannon) than those of women who were normal weight or obese prior to becoming pregnant (Table 3). There was no difference in the alpha diversity of infant fecal bacterial communities by maternal pre-pregnancy BMI (Table 3). Alpha diversity compared by infant age, sex, mode of delivery, breastfeeding exclusivity, antibiotic use since birth, and sample shipping time were not significant; however, the ARCH_GUT_ infants had a significantly higher richness compared to the BABY_GUT_ cohort (S1 Table). After stratifying the infants by maternal pre-pregnancy BMI and removing infants exposed to antibiotics since birth (n = 4), those in the obese category that were breastfed exclusively had lower richness and Shannon diversity scores compared to non-exclusively breastfed infants (S2 Table). Alpha diversity of the infant fecal microbiota did not differ by mode of delivery for women who were overweight or obese prior to becoming pregnant. No comparisons between delivery mode and breastfeeding were possible in the normal weight group because of a lack of c-section deliveries and infants fed a mixed diet (n = 1 for both).

**Table 3:** Alpha diversity of the fecal microbiota of mothers and infants by maternal Pre-pregnancy BMI category.

Pregnant Women^1^	All	Normal	Overweight	Obese	p-value
Chao1	122.7 ± 23.0	133.3 ± 24.9^a^	107.7 ± 18.9^b^	125.1 ± 19.7^ab^	0.02
Inverse Simpson	9.6 ± 4.7	10.9 ± 5.4	6.7 ± 5.0	10.6 ± 3.1	0.05
Shannon	2.8 ± 0.5	3.0 ± 0.4^a^	2.4 ± 0.7^b^	2.9 ± 0.3^a^	0.02
**Infants**^**1**^					
Chao1	46.3 ± 20.6	49.5 ± 22.0	44.2 ± 11.4	45.4 ± 25.0	0.58
Inverse Simpson	3.3 ± 1.2	3.7 ± 1.5	3.0 ± 0.9	3.2 ± 1.1	0.49
Shannon	1.4 ± 0.4	1.5 ± 0.4	1.4 ± 0.3	1.4 ± 0.4	0.51

Values in a row that do not contain the same superscript are significantly different, p<0.05

^1^Values reported as mean ± SD

### Beta diversity

Pregnant women had different bacterial communities than infants at both the genus and phylum levels for both Sorensen and Bray-Curtis dissimilarities (p = 0.001) (S1 Fig). The dispersion was also significantly different at the genus and phylum levels (p = 0.001) due to the large variation in infant fecal bacterial composition and the relatively more similar communities in the women.

**Fig 1:**
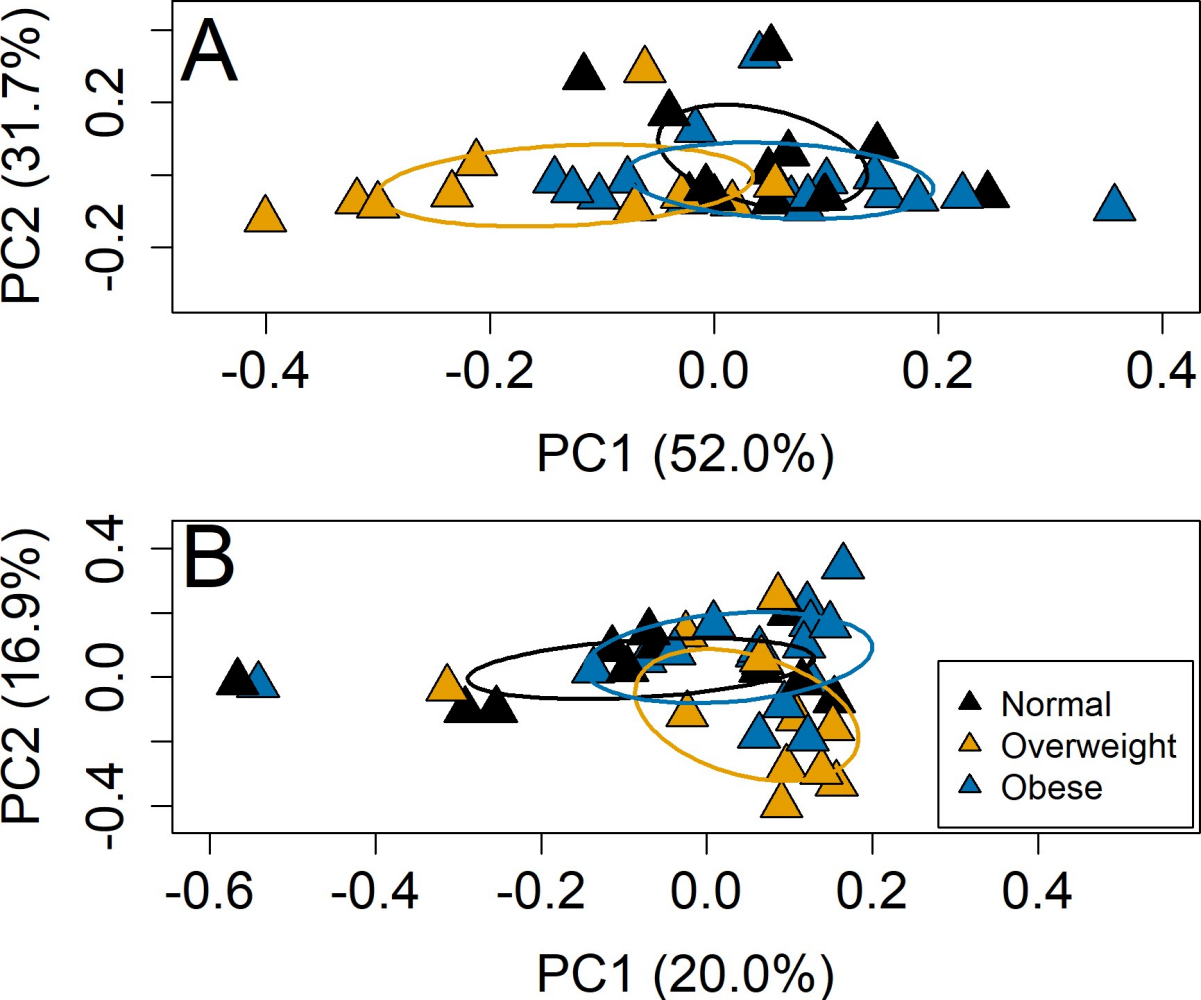
Pre-pregnancy overweight women have different fecal microbiota compositions than normal and obese women at phylum/genus levels. PCoA of the Bray-Curtis dissimilarity at the (A) phylum-level and (B) genus-level. Axes percentages represent the amount of variation in the data explained by the axis, calculated from the PCoA eigen values. Axes ranges represent the relative dissimilarity present between the samples.

We separately assessed changes in community composition (accounting for the presence and absence of taxa based on Sorensen’s index) and community structure (accounting for the relative contributions of taxa based on Bray-Curtis dissimilarity) in the pregnant women and infants. During pregnancy, the fecal bacterial community structure of overweight women differed by BMI category at the phylum (F = 4.1, p = 0.003) and genus levels (F = 1.7, p = 0.02) (Fig 1A and 1B). Adjusting for maternal age, cohort and shipping time, BMI category remained significant at the phylum (F = 3.9, p = 0.003) and genus level (F = 1.6, p = 0.03) for community structure. In addition, cohort (F = 2.1, p = 0.02) and shipping time (F = 1.8, p = 0.04) were significantly associated with the fecal bacterial communities at the genus level. The overall model for maternal age, cohort, shipping time and BMI category was significant at the phylum (F = 2.32, p = 0.01) and genus levels (F = 1.7, p = 0.001). There were no differences by BMI category or any other variables for community composition by the Sorensen index (S2 Fig).

**Fig 2:**
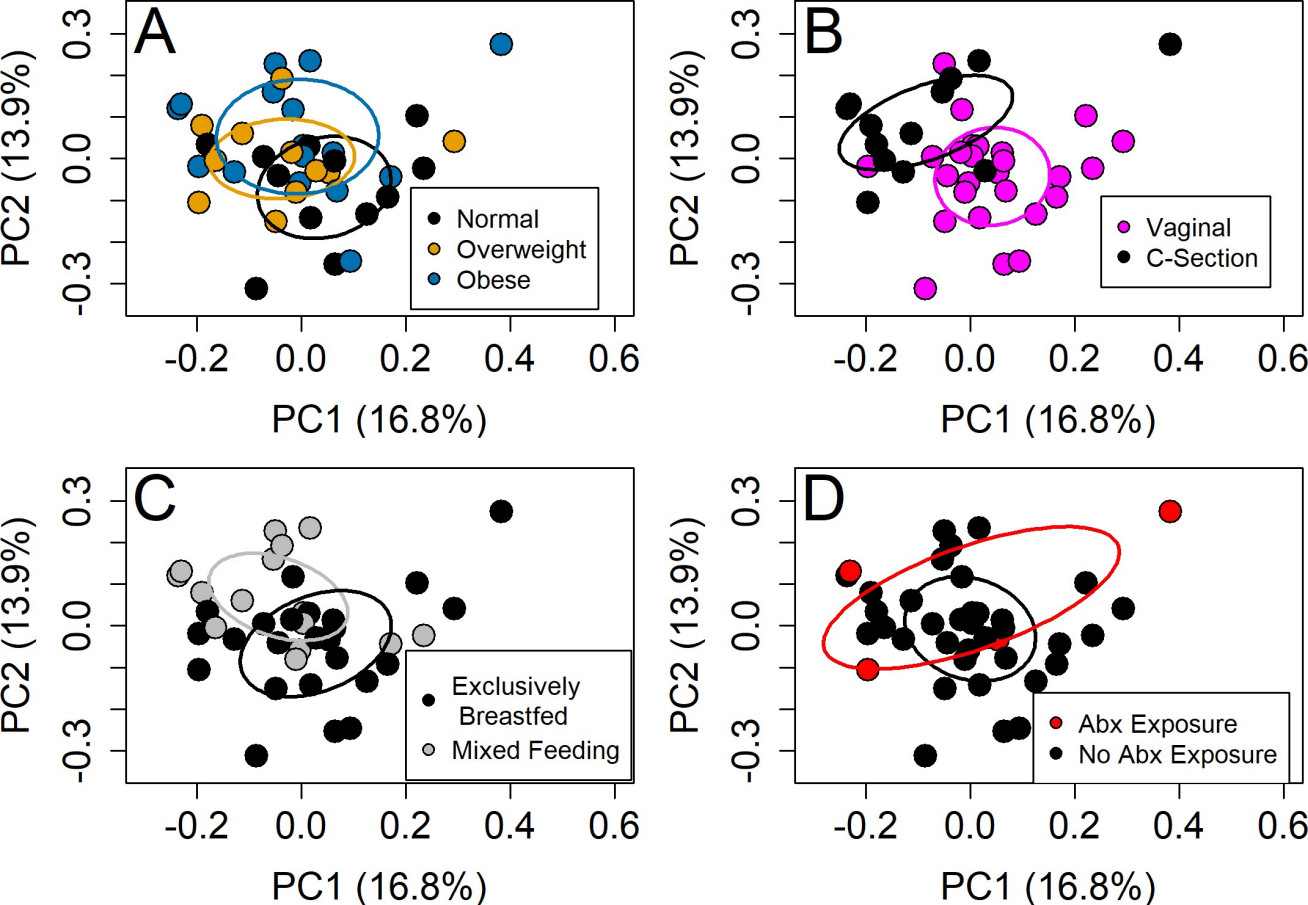
Relationship between infant fecal bacterial membership and maternal/infant variables at the genus level. PCoA of the genus-level microbiota using the Sorensen index comparing (A) maternal pre-pregnancy BMI category, (B) delivery mode, (C) breast milk in diet and (D) antibiotic exposure. Axes percentages represent the amount of variation in the data explained by the axis, calculated from the PCoA eigen values. Axes ranges represent the relative dissimilarity present between the samples.

In univariate analyses, infant community composition (Sorensen index) at the genus level tended to differ by maternal pre-pregnancy BMI Category (F = 1.4, p = 0.06) and significantly differed by delivery mode (F = 2.9, p = 0.001) and breastmilk in the infant diet (F = 2.2, p = 0.008) (Fig 2). In a multivariate model including BMI, breastfeeding, delivery mode, sex and antibiotic exposure, delivery mode was significant using the Sorensen index (F = 2.1, p = 0.01), and exposure to antibiotics was significant for Bray-Curtis (F = 2.1, p = 0.04). After additionally adjusting for sample shipping time, sex and cohort, none of the variables were significant for Sorensen. In univariate analyses, antibiotic exposure was significant for Bray-Curtis (F = 2.2, p = 0.03), but maternal pre-pregnancy BMI category was not associated with infant gut community structure (Bray-Curtis dissimilarity matrix, S3 Fig). Similarly, the overall multivariate model for community structure (Bray-Curtis dissimilarity matrix) of the infant microbiota was not significant. At the phylum level, there were no significant differences.

When stratifying the infant samples by maternal pre-pregnancy BMI category and removing infants exposed to antibiotics (n = 4), only breastfeeding was associated with the Sorensen index at the genus level in the subset of infants born to women who were obese prior to becoming pregnant (F = 2.0, p = 0.02) (S4 Fig). At the phylum level, breastfeeding was significantly associated with the gut microbiota community in infants born to either overweight (F = 2.7, p = 0.048) or obese (F = 3.7, p = 0.009) women (S4 Fig).

### Sensitivity analysis of infant microbiota data

Since age is known to be associated with changes in the infant microbiota [4, 10, 44], we conducted a sensitivity analysis on two subsets of the infant data based on the median age (8.5 days) and the mean age (18 days) of the infants. Alpha diversity in either age subset was not associated with maternal pre-pregnancy BMI, delivery mode, age, sex, sample shipping time or cohort (S3 Table). For infants ≤9 days of age, only infant sex significantly associated with gut microbiota membership as measured by the Sorensen metric (F = 1.8, 0.03), however, the dispersion was also significantly different (F = 5.6, p = 0.03) (S5 Fig). For infants ≤18 days of age, the Sorensen metric was significant by maternal BMI (F = 1.5, p = 0.04) and delivery mode (F = 1.92, p = 0.03) (S6 Fig).

### Differential patterns of bacterial taxa

Women who were overweight prior to becoming pregnant had higher abundances of *Bacteroides* and lower abundances of *Phascolarctobacterium* than women who were normal weight or obese prior to becoming pregnant. Women who were normal weight prior to becoming pregnant had lower abundances of *Acidaminococcus* and *Dialister* but higher abundances of *Phascolarctobacterium* than overweight and obese women (Table 4). At the phylum level, the overweight women had higher Bacteroidetes than both normal weight and obese women, but lower abundances of Firmicutes than obese women (Table 4).

**Table 4:** Significantly different taxa in the fecal microbiota by maternal pre-pregnancy BMI category.

**Pregnant Women—Genus**	Normal	Overweight	Obese
Bacteroides	20.0 ± 7.2^b^	38.1 ± 21.7^a^	19.8 ± 8.2^b^
Phascolarctobacterium	3.0 ± 3.1^a^	1.7 ± 2.3^c^	2.3 ± 3.1^b^
Acidaminococcus	0.0006 ± 0.002^b^	2.4 ± 6.7^a^	1.9 ± 4.8^a^
Dialister	0.7 ± 1.0^c^	1.1 ± 1.6^b^	1.7 ± 2.6^a^
**Pregnant Women—Phylum**			
Bacteroidetes	29.5 ± 8.8^b^	49.3 ± 17.6^a^	30.2 ± 12.1^b^
Firmicutes	49.5 ± 11.1^ab^	37.6 ± 15.0^b^	52.7 ± 14.0^a^
**Infants—Genus**			
Megasphaera	0.09 ± 0.2^b^	6.0 ± 19.0^a^	9.5 ± 21.5^a^
Streptococcus	7.0 ± 9.2^a^	0.9 ± 1.2^b^	2.6 ± 4.1^ab^
Staphylococcus	5.3 ± 9.4^a^	2.1 ± 2.5^ab^	0.7 ± 1.4^b^
Acidaminococcus	0.0006 ± 0.002^b^	0.0006 ± 0.002^b^	3.4 ± 13.6^a^
Escherichia-Shigella	28.1 ± 28.3^a^	19.9 ± 20.1^b^	17.7 ± 24.1^c^
Akkermansia	0.004 ± 0.004^b^	0.006 ± 0.009^b^	2.6 ± 9.7^a^
**Infants—Phylum**			
Verrucomicrobia	0.005 ± 0.004^b^	0.005 ± 0.008^b^	2.6 ± 9.7^a^

Values reported as mean (%) ± SD

Values in a row that do not contain the same superscript are significantly different, p<0.05

P-values are Benjamini-Hochberg corrected

Infants born to women who were normal weight prior to becoming pregnant had lower abundances of *Megasphaera*, but higher abundances of *Escherichia-Shigella* than infants born to overweight or obese women. *Streptococcus* was less abundant in infants from women who were overweight prior to pregnancy, and *Staphylococcus* was lower in infants from obese women (Table 4). Infants born to obese women had significantly more *Acidaminococcus* and *Akkermansia* in their fecal bacterial communities than infants born to normal or overweight women. Vaginally-born infants had significantly higher abundances of *Megasphaera*, *Parabacteroides*, and *Escherichia-Shigella*, but lower *Acidaminococcus* and *Akkermansia* than C-section born infants (Table 5). Infants that exclusively consumed human milk had higher abundances of *Staphylococcus* and *Escherichia-Shigella*, but lower abundances of *Acidaminococcus* and *Akkermansia* than infants that consumed a mixed diet (Table 5).

**Table 5:** Significantly different taxa in fecal microbiota by delivery mode and breastfeeding.

**Infant—Genus**	Vaginal	C-section
Megasphaera	8.4 ± 20.5	0.009 ± 0.02
Parabacteroides	4.8 ± 10.8	0.3 ± 0.9
Escherichia-Shigella	22.4 ± 26.4	19.9 ± 20.0
Akkermansia	0.01 ± 0.03	3.1 ± 10.8
Acidaminococcus	0.0005 ± 0.002	4.2 ± 15.1
**Infant—Phylum**		
Verrucomicrobia	0.01 ± 0.03	3.1 ± 10.8
**Infant—Genus**	Exclusively Breastfed	Mixed Feeding
Escherichia-Shigella	24.1 ± 24.1	17.4 ± 18.2
Akkermansia	0.07 ± 0.3	2.6 ± 10.1
Acidaminococcus	0.002 ± 0.006	3.6 ± 14.1
Staphylococcus	3.8 ± 6.9	0.4 ± 1.0
**Infant—Phylum**		
Verrucomicrobia	0.08 ± 0.3	2.6 ± 10.1
**Infant—Genus**	Antibiotics Since Birth	No Antibiotics
Megasphaera	0.04 ± 0.04	6.2 ± 18.0
Parabacteroides	0.01 ± 0.02	3.6 ± 9.5
Escherichia-Shigella	0.15 ± 0.3	24.0 ± 24.4
Acidaminococcus	0.007 ± 0.01	1.6 ± 9.2
**Infant—Phylum**		
Bacteroidetes	0.9 ± 1.0	13.7 ± 20.4

Values reported as mean (%) ± SD

All comparisons, p<0.05 (Benjamini-Hochberg corrected)

After stratifying the infant data by BMI category, infants born vaginally to women who were overweight prior to becoming pregnant had higher abundances of *Bifidobacterium*, *Megasphaera and Parabacteroides* but lower abundances of unclassified *Enterobacteriaceae*, *Clostridium sensu stricto*, *Escherichia-Shigella*, and *Enterococcus* (S4 Table). Because 4 of the 5 infants born vaginally to overweight women were exclusively breastfed, the same associations were observed when comparing exclusively breastfed and mixed diet infants. In the obese group, *Megasphaera* and *Clostridium sensu stricto* were more abundant, and *Akkermansia* and *Klebsiella* were less abundant in the vaginally delivered infants compared to the c-section delivered infants (S5 Table). In contrast to babies born vaginally to women who were overweight prior to becoming pregnant, the infants born vaginally to women who were obese prior to becoming pregnant were just as likely to be mixed fed as they were to be exclusively breastfed. Infants born to women who were obese prior to becoming pregnant and fed a diet of exclusive breastmilk had higher abundances of *Staphylococcus*, but lower abundances of *Megasphaera*, *Akkermansia* and *Klebsiella* than infants fed a mixed diet. At the phylum level, the gut microbiota of infants born to women that were obese prior to becoming pregnant had a greater abundance of Verrucomicrobia when delivered by c-section or fed a mixed diet (S5 Table).

## Discussion

In this population of pregnant women and infants, we investigated the relationship between maternal pre-pregnancy BMI and the gut microbiota of women during late pregnancy and of their children during early infancy. The fecal bacterial communities of women who were overweight prior to becoming pregnant differed from those of women who were normal weight and obese prior to becoming pregnant. The overweight women had a lower alpha diversity and a high abundance of *Bacteroides*, which was likely the main driver of their overall community differences measured by beta diversity, compared to the other BMI categories. The alpha diversity of the infant microbiota did not differ by any of variables tested, but the beta diversity of the infant microbiota was associated with delivery mode, human milk in the diet and antibiotic exposure when analyzed independently. However, only exclusive breastfeeding was significantly associated with the infant gut microbiota after stratifying by maternal pre-pregnancy BMI category.

Women who were overweight prior to becoming pregnant tended to have a different microbiota than normal weight and obese women. Although some studies have found no differences in the gut communities of normal, overweight or obese women during pregnancy [10, 45], our population of pre-pregnancy overweight women had a different community structure at the genus and phylum levels than normal and obese women. This difference was driven by the higher abundance of *Bacteroides* and Bacteroidetes in the feces of overweight women compared to that of women in the other BMI categories. That the third trimester microbiota of overweight women was different from the third trimester microbiota of normal and obese women was surprising. A higher abundance of *Bacteroides* in obese women compared to normal weight has been observed previously using fluorescent in-situ hybridization [19]; however, there were no participants in the overweight category as reference. Our population of overweight women also had microbiota with lower richness, lower diversity and higher evenness than that of normal weight or obese women as well as a significantly different community composition. Our results in overweight pregnant women may be due to other variables related to BMI, such as diet or lifestyle [46], which have also been shown to alter the microbiome. Another possible reason for this difference could be gestational weight gain. Excessive gestational weight gain has been associated with a gut microbiota dominated by Bacteroidetes rather than Firmicutes and a lower alpha diversity [20]. Thus, the overweight women herein may have had a different microbiota because of higher gestational weight gain compared to the normal weight or obese women. Unfortunately, this information was not collected. Both gestational weight gain and maternal BMI have been associated with differences in the child’s gut microbiota between 4 days and 2 years of age [47]. Shifts in gut microbiota composition over the course of pregnancy due to hormonal, metabolic and immunological changes [16] may affect what taxa establish in the infant gut. Some bacteria found in the infant gut match bacteria found in their mother’s gut at the strain level [48, 49] suggesting there is a partial transfer of gut bacteria from mother to child [22].

Since the microbiota influences weight gain and there is evidence of microbial transmittance from mother to child, it is possible an obesogenic microbiota can be transferred from mother to child. This, in turn, may increase the child’s risk of developing obesity. In our infant population, we found that microbiota membership was affected by both maternal characteristics and environmental exposures. Delivery mode and amount of breast milk in the diet were associated with the infant fecal microbiota before adjustment, while maternal pre-pregnancy BMI tended to be associated with the infant fecal microbiota. Many of the bacterial abundances that differed by maternal pre-pregnancy BMI, delivery mode and breast milk in the diet were shared, such as *Escherichia-Shigella* and *Acidaminococcus*. In children that are at risk for malnourishment, *Acidaminococcus* has been shown to negatively affect growth [50]. These associations suggest that maternal pre-pregnancy BMI category, delivery mode and amount of breast milk in the diet all interact in similar ways with the infant microbiota. This may be due to associations between the pre-pregnancy BMI, delivery mode and breastfeeding variables themselves. For example, infants born to overweight and obese women have a higher risk of developing childhood overweight/obesity and a 2–4 fold increased odds of being delivered via C-section compared to normal weight women [51–53]. Moreover, obese mothers often have difficulties breastfeeding due to insufficient milk yields in the first few weeks of lactation [54] and are more likely to give their infant formula rather than breastmilk as a result [54, 55].

After adjusting for maternal pre-pregnancy BMI, shipping time, infant age, breast milk and mode of delivery, delivery mode remained significantly associated with the beta-diversity (Sorenson index) of the infant gut microbiota. Additionally, vaginally-born infants had higher abundance of *Parabacteroides* and *Escherichia-Shigella*. Others have found that vaginally born infants have a higher proportion of their community in common with their mothers’ microbiota such as *Bifidobacterium* and *Parabacteroides* and *Escherichia-Shigella* [4]. *Bifidobacterium* was significantly higher in our vaginally-born infants only in the overweight, but not obese, category. This was related to the disparities in breastfeeding exclusivity between infants born to overweight women versus those born to obese women with each mode of delivery category. Regardless of maternal pre-pregnancy BMI status, 33.3% of c-section born infants were exclusively fed human milk. In contrast, 80% of vaginally born infants of overweight women and 50% of vaginally born infants of obese women were exclusively fed human milk. Infants born to obese women, even if they are vaginally born, may be at a greater risk for allergies, overweight/obesity and other chronic diseases if their gut microbiota lack of *Bifidobacteria* [56].

Breastfeeding impacts the abundances of several taxa, especially the genera *Lactobacillus* and *Bifidobacteria*, which can utilize the oligosaccharides found in breast milk [4, 28, 29, 57]. Breastfeeding was not associated with the alpha- or beta-diversity of the infant microbiota after adjustment for covariates, but breastfeeding was significantly associated with community membership at the genus-level and composition at the phylum-level when stratifying by maternal pre-pregnancy BMI. Compared to the relationship we found between delivery mode and the microbiota, we saw similar associations between BMI category and *Bifidobacterium* abundance when comparing exclusively breastfed infants to mixed-fed infants. However, this is likely due to collinearity between delivery mode and breastfeeding within the overweight group [4]. Others have shown that exclusive breastfeeding alters the microbiota compared to mixed feeding [4, 58] and reduces the risk of overweight/obesity in both childhood and adulthood [59].

Infant age is associated with changes to the microbiota for several reasons: alterations to the gut environment, allowing for a shift from a high abundance of facultative anaerobes to obligate anaerobes over time; alterations in dietary intake; and alterations in gut transit time [44]. The full analysis and the sensitivity analyses of infants 9 days old or less and infants 18 days old or less led to similar conclusions for alpha diversity in infant fecal bacterial communities. Although, beta-diversity results were similar for the full population and the subset of infants 2–18 days of age, these results were not similar for the subset of infants 2–9 days of age. This is likely due to decreasing sample size which reduced the number of infants in the obese category to 31%, those in the overweight category to 45% and those in the normal category to 75% of their original size.

There are several limitations to this study. One limitation is the sample collection method. Women collected the samples in their home and shipped the samples to the lab allowing the sample to remain at non-ideal temperatures for an average of 4 days before storage at -80°C. In general, it is known that the methods used to collect, store, extract and amplify samples have effects on microbiome data [60–62]. These conditions may affect the relative abundances of the microbiota, but community differences across samples have been shown to be preserved regardless of storage method [61, 63, 64]. Thus, comparisons across groups within our study population are valid. The BMI and breastfeeding variables relied on self-reported data from the women. The pre-pregnancy self-report of height and weight has been reported to be valid [39], but other studies have found that self-reported height and weight can be prone to error [65]. The measure of breastmilk in the infant diet also relied on self-report [40], so there may be measurement errors in the reported values. Furthermore, because of the relatively limited sample size and collinearity among variables, we were not able to test some associations, such as breastfeeding exclusivity and mode of delivery within the normal weight participants, in this data set. For instance, if an infant was born vaginally, s/he was also likely to be exclusively fed human milk in this study population. Antibiotic use by the infant was included in the analysis because antibiotic use has been shown to alter the gut microbiota [66]. However, we did not include maternal antibiotic use during pregnancy or parturition in this analysis, and these exposures may also impact the pregnancy and infancy gut microbiotas. Strengths of this study include the enrollment of participants of low socioeconomic status, a factor which has been shown to reduce gut microbial diversity in adults [67].

## Conclusion

Maternal pre-pregnancy BMI is associated with the pregnancy fecal bacterial community and tends to be associated with the early infancy fecal bacterial community. Other maternal characteristics and environmental exposures were also associated with the microbiota during early infancy. Factors such as pre-pregnancy BMI, C-section delivery and formula feeding, affect the infant microbiota and have also been shown to increase the risk of developing adverse health outcomes such as childhood overweight/obesity [68] and allergies/asthma [69–71]. The health effects associated with these factors may partially be explained by their effects on the microbiota. Other characteristics of the infant microbiome, such as species, strain, or functional differences, are important aspects of microbiota-host interactions and may give further insight on the roles the microbiota plays in childhood development. Future work will determine if these bacterial differences persist as the child ages as well as describe associations between the microbiota and later health outcomes.

## Supporting information

S1 Table Infant fecal microbiota alpha diversity by infant age, sex, mode of delivery and sample shipping time. (PDF)

S2 Table P-values of infant fecal microbiota alpha diversity by delivery mode and breastfeeding stratified by BMI category. (PDF)

S3 Table Sensitivity of infant fecal microbiota alpha diversity to infant age. (PDF)

S4 Table Significantly different taxa by delivery mode and breastfeeding in the gut microbiota of infants born to women who were overweight prior to becoming pregnant. (PDF)

S5 Table Significantly different taxa by delivery mode and breastfeeding in the gut microbiota of infants born to women who were obese prior to becoming pregnant. (PDF)

S1 Fig The fecal bacterial community structure of pregnant women differs significantly from that of infants. PCoA of the microbiota for all samples at the (A) phylum-level using Bray-Curtis dissimilarity, and at the genus-level using (B) Sorensen index and (C) Bray-Curtis Dissimilarity. Axes percentages represent the amount of variation in the data explained by the axis, calculated from the PCoA eigen values. Axes ranges represent the relative dissimilarity present between the samples.
(TIF)

S2 Fig Bacterial community membership does not differ in pregnant women by pre-pregnancy BMI category. PCoA of the genus-level microbiota using Sorensen index. Axes percentages represent the amount of variation in the data explained by the axis, calculated from the PCoA eigen values. Axes ranges represent the relative dissimilarity present between the samples.
(TIF)

S3 Fig Relationship between infant fecal bacterial structure at the genus level and maternal/infant variables. PCoA of the genus-level microbiota by Bray-Curtis dissimilarity comparing (A) maternal pre-pregnancy BMI category, (B) delivery mode, (C) breast milk in diet and (D) antibiotic exposure. Axes percentages represent the amount of variation in the data explained by the axis, calculated from the PCoA eigen values. Axes ranges represent the relative dissimilarity present between the samples.
(TIF)

S4 Fig Infant fecal microbiota stratified by maternal overweight and obese categories. PCoA of the infant gut microbiota by breastfeeding status of the (A) overweight group using the Sorensen index at the genus level (B) obese group using the Sorensen index at the genus level (C) overweight group using Bray-Curtis dissimilarity at the phylum level (D) obese group using Bray-Curtis dissimilarity at the phylum level. Axes percentages represent the amount of variation in the data explained by the axis, calculated from the PCoA eigen values. Axes ranges represent the relative dissimilarity present between the samples.
(TIF)

S5 Fig Sensitivity analysis of the infant microbiota, including infants from 2 to 9 days old. PCoA of the genus-level microbiota by Sorensen dissimilarity comparing (A) maternal pre-pregnancy BMI category, (B) delivery mode, (C) breastfeeding status and (D) sex. Axes percentages represent the amount of variation in the data explained by the axis, calculated from the PCoA eigen values. Axes ranges represent the relative dissimilarity present between the samples.
(TIF)

S6 Fig Sensitivity analysis of the infant microbiota, including infants from 2 to 18 days old. PCoA of the genus-level microbiota by Sorensen dissimilarity comparing (A) maternal pre-pregnancy BMI category, (B) delivery mode, (C) breastfeeding status and (D) sex. Axes percentages represent the amount of variation in the data explained by the axis, calculated from the PCoA eigen values. Axes ranges represent the relative dissimilarity present between the samples.
(TIF)
