# Supplementary material for: Michigan cohorts to determine associations of maternal pre-pregnancy body mass index with pregnancy and infant gastrointestinal microbial communities: Late pregnancy and early infancy
Source: PLoS One. 2019 Mar 18;14(3):e0213733. doi: 10.1371/journal.pone.0213733 (PMC6422265; doi:10.1371/journal.pone.0213733)
Supplement: S1 Table — (PDF) [file pone.0213733.s001.pdf]

Age and Shipping[1]	Infant Age	Shipping Time	
Chao1	ρ=-0.06, p=0.72	ρ=-0.01, p=0.93	
Inverse Simpson	ρ=-0.08, p=0.63	ρ=-0.13, p=0.45	
Shannon	ρ=-0.005, p=0.98	ρ=-0.13, p=0.43	
**Delivery Mode[2]**	**Vaginal**	**C-Section**	**p-value**
Chao1	47.0 ± 16.2	44.9 ± 28.2	0.16
Inverse Simpson	3.3 ± 1.4	3.2 ± 0.8	0.8
Shannon	1.4 ± 0.4	1.5 ± 0.3	0.65
**Sex[2]**	**Males**	**Females**	
Chao1	49.0 ± 24.7	41.5 ± 8.6	0.63
Inverse Simpson	3.2 ± 1.3	3.4 ± 1.2	0.65
Shannon	1.5 ± 0.4	1.4 ± 0.4	0.78
**Cohort[2]**	**Baby**	**ARCH**	
Chao1	51.0 ± 23.9	38.8 ± 10.9	0.03
Inverse Simpson	3.6 ± 1.4	2.9 ± 0.9	0.14
Shannon	1.5 ± 0.4	1.3 ± 0.3	0.05
**Breastfeeding[2]**	**Exclusive**	**Mixed**	
Chao1	48.4 ± 24.6	43.0 ± 12.0	0.98
Inverse Simpson	3.2 ± 1.4	3.3 ± 1.4	0.48
Shannon	1.4 ± 0.4	1.5 ± 0.3	0.45
**Antibiotic Used Since Birth[2]**	**Any**	**None**	
Chao1	56.8 ± 51.8	45.1 ± 14.9	0.39
Inverse Simpson	2.6 ± 0.5	3.4 ± 1.3	0.19
Shannon	1.3 ± 0.4	1.5 ± 0.4	0.46

[1]rho and p-values reported

[2]mean ± SD
