# Supplementary material for: Michigan cohorts to determine associations of maternal pre-pregnancy body mass index with pregnancy and infant gastrointestinal microbial communities: Late pregnancy and early infancy
Source: PLoS One. 2019 Mar 18;14(3):e0213733. doi: 10.1371/journal.pone.0213733 (PMC6422265; doi:10.1371/journal.pone.0213733)
Supplement: S2 Table — (PDF) [file pone.0213733.s002.pdf]

Normal	**ROUT**	**BF**
Chao1	**NA**	**NA**
Inverse Simpson	**NA**	**NA**
Shannon	**NA**	**NA**
Overweig	**ROUT**	**BF**
Chao1	**0.19**	**0.73**
Inverse Simpson	**0.41**	**1**
Shannon	**0.73**	**0.73**
Obese	**ROUT**	**BF**
Chao1	**0.78**	**0.04**
Inverse Simpson	**0.3**	**0.23**
Shannon	**0.45**	**0.03**

p-values reported
