# Supplementary material for: Michigan cohorts to determine associations of maternal pre-pregnancy body mass index with pregnancy and infant gastrointestinal microbial communities: Late pregnancy and early infancy
Source: PLoS One. 2019 Mar 18;14(3):e0213733. doi: 10.1371/journal.pone.0213733 (PMC6422265; doi:10.1371/journal.pone.0213733)
Supplement: S3 Table — (PDF) [file pone.0213733.s003.pdf]

Infants ≤ 9 Days of Age				
**Age and Shipping**[1]	**Infant Age**		**Shipping Time**	
Chao1	ρ=-0.26, p=0.29		ρ=-0.06, p=0.81	
Inverse Simpson	ρ=-0.18, p=0.46		ρ=0.11, p=0.65	
Shannon	ρ=-0.26, p=0.29		ρ=0.02, p=0.94	
	**Normal**	**Overweight**	**Obese**	**p-value**
n	9	5	5	
Chao1	50.8 ± 24.6	51.8 ± 12.0	37.2 ± 6.9	0.2
Inverse Simpson	3.8 ± 1.7	3.1 ± 1.1	2.5 ± 0.6	0.41
Shannon	1.5 ± 0.5	1.6 ± 0.4	1.2 ± 0.2	0.53
**Delivery Mode**[2]		**Vaginal**	**C-Section**	
Chao1		48.7 ± 20.5	42.9 ± 10.7	0.66
Inverse Simpson		3.3 ± 1.5	3.4 ± 1.0	0.47
Shannon		1.4 ± 0.4	1.5 ± 0.4	0.74
**Sex**[2]		**Males**	**Females**	
Chao1		49.3 ± 21.0	42.5 ± 10.5	0.89
Inverse Simpson		3.3 ± 1.5	3.3 ± 1.3	0.69
Shannon		1.4 ± 0.4	1.4 ± 0.4	0.89
**Cohort**[2]		**Baby**	**ARCH**	
Chao1		38.7 ± 10.7	53.9 ± 21.2	0.06
Inverse Simpson		2.9 ± 1.0	3.6 ± 1.6	0.54
Shannon		1.3 ± 0.3	1.5 ± 0.4	0.18
**Breastfeeding**[2]		**Exclusive**	**Mixed**	
Chao1		47.9 ± 21.1	46.7 ± 14.3	1
Inverse Simpson		3.5 ± 1.6	2.8 ± 0.7	0.77
Shannon		1.4 ± 0.5	1.4 ± 0.25	0.97

Infants ≤ 18 Days of Age				
**Age and Shipping**[1]	**Infant Age**		**Shipping Time**	
Chao1	ρ=-0.25, p=0.22		ρ=-0.04, p=0.86	
Inverse Simpson	ρ=0.01, p=0.94		ρ=-0.10, p=0.63	
Shannon	ρ=-0.03, p=0.88		ρ=-0.19, p=0.35	
	**Normal**	**Overweight**	**Obese**	**p-value**
n	10	7	8	
Chao1	50.4 ± 23.3	48.4 ± 11.8	37.8 ± 6.6	0.2
Inverse Simpson	3.8 ± 1.6	3.1 ± 1.0	2.9 ± 1.1	0.59
Shannon	1.5 ± 0.5	1.4 ± 0.3	1.3 ± 0.4	0.57
**Delivery Mode**[2]		**Vaginal**	**C-Section**	
Chao1		46.9 ± 18.6	42.3 ± 9.2	0.73
Inverse Simpson		3.2 ± 1.4	3.7 ± 1.0	0.22
Shannon		1.4 ± 0.4	1.6 ± 0.3	0.33
**Sex**[2]		**Males**	**Females**	
Chao1		48.1 ± 19.2	40.9 ± 9.1	0.51
Inverse Simpson		3.4 ± 1.5	3.3 ± 1.1	0.84
Shannon		1.5 ± 0.4	1.4 ± 0.4	0.67
**Cohort**[2]		**Baby**	**ARCH**	
Chao1		39.4 ± 10.2	49.4 ± 18.9	0.11
Inverse Simpson		2.8 ± 1.0	3.7 ± 1.4	0.21
Shannon		1.3 ± 0.3	1.5 ± 0.4	0.1
**Breastfeeding**[2]		**Exclusive**	**Mixed**	
Chao1		46.0 ± 18.8	45.3 ± 12.8	0.93
Inverse Simpson		3.4 ± 1.5	3.2 ± 0.9	0.98
Shannon		1.4 ± 0.4	1.5 ± 0.3	0.8

[1]rho and p-values reported

[2]mean ± SD
