# Supplementary material for: Michigan cohorts to determine associations of maternal pre-pregnancy body mass index with pregnancy and infant gastrointestinal microbial communities: Late pregnancy and early infancy
Source: PLoS One. 2019 Mar 18;14(3):e0213733. doi: 10.1371/journal.pone.0213733 (PMC6422265; doi:10.1371/journal.pone.0213733)
Supplement: S4 Table — (PDF) [file pone.0213733.s004.pdf]

| **Infant - Genus** | Vaginal | C-section |
|---|---|---|
| Uncl. Enterobacteriaceae | 3.6 ± 1.7 | 17.8 ± 26.0 |
| Bifidobacterium | 27.7 ± 26.8 | 11.4 ± 11.4 |
| Megasphaera | 16.6 ± 31.1 | 0.004 ± 0.006 |
| Parabacteroides | 2.9 ± 3.9 | 0.6 ± 1.5 |
| Clostridium Sensu Stricto | 0.01 ± 0.02 | 16.9 ± 24.0 |
| Escherichia-Shigella | 17.6 ± 23.0 | 30.0 ± 17.0 |
| Enterococcus | 0.04 ± 0.04 | 1.7 ± 2.3 |
| **Infant - Genus** | Exclusively Breastfed | Mixed Feeding |
| Uncl. Enterobacteriaceae | 3.3 ± 1.4 | 18.7 ± 25.8 |
| Bifidobacterium | 30.1 ± 25.3 | 9.5 ± 10.0 |
| Megasphaera | 16.6 ± 31.1 | 0.005 ± 0.006 |
| Parabacteroides | 2.9 ± 3.9 | 0.6 ± 1.4 |
| Veillonella | 0.2 ± 0.4 | 1.7 ± 1.9 |
| Clostridium Sensu Stricto | 0.01 ± 0.02 | 16.9 ± 23.9 |
| Escherichia-Shigella | 13.9 ± 16.8 | 32.6 ± 19.1 |
| Staphylococcus | 2.8 ± 3.1 | 1.2 ± 1.5 |
| Enterococcus | 0.1 ± 0.2 | 1.6 ± 2.4 |
