# Supplementary material for: Michigan cohorts to determine associations of maternal pre-pregnancy body mass index with pregnancy and infant gastrointestinal microbial communities: Late pregnancy and early infancy
Source: PLoS One. 2019 Mar 18;14(3):e0213733. doi: 10.1371/journal.pone.0213733 (PMC6422265; doi:10.1371/journal.pone.0213733)
Supplement: S5 Table — (PDF) [file pone.0213733.s005.pdf]

**Infant - Genus**	Vaginal	C-section
Megasphaera	15.1 ± 26.0	0.002 ± 0.003
Clostridium sensu stricto	13.1 ± 17.2	0.7 ± 0.2
Akkermansia	0.02 ± 0.05	9.8 ± 19.5
Klebsiella	0.5 ± 0.8	9.1 ± 10.6
**Infant - Phylum**		
Verrucomicrobia	0.02 ± 0.05	9.8 ± 19.5
**Infant - Genus**	Exclusively Breastfed	Mixed Feeding
Megasphaera	0.3 ± 0.6	18.7 ± 28.2
Staphylococcus	10.9 ± 16.8	8.5 ± 15.5
Akkermansia	0.004 ± 0.003	4.9 ± 13.8
Klebsiella	0.5 ± 1.0	4.8 ± 8.3
**Infant - Phylum**		
Verrucomicrobia	0.006 ± 0.005	4.9 ± 13.8
