# Supplementary figures and images for: Michigan cohorts to determine associations of maternal pre-pregnancy body mass index with pregnancy and infant gastrointestinal microbial communities: Late pregnancy and early infancy
Source: PLoS One. 2019 Mar 18;14(3):e0213733. doi: 10.1371/journal.pone.0213733 (PMC6422265; doi:10.1371/journal.pone.0213733)

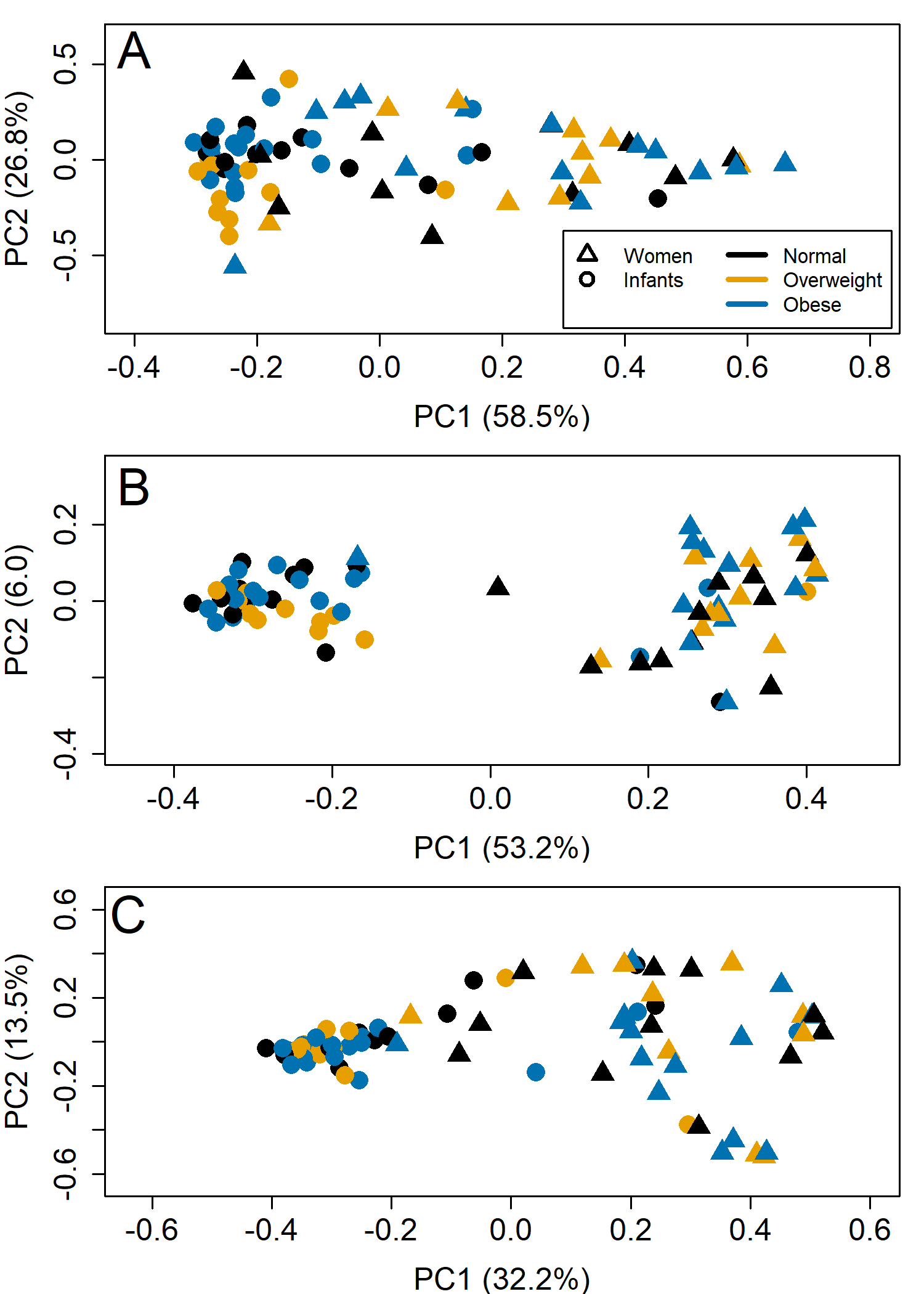

Supplement: S1 Fig — PCoA of the microbiota for all samples at the (A) phylum-level using Bray-Curtis dissimilarity, and at the genus-level using (B) Sorensen index and (C) Bray-Curtis Dissimilarity. Axes percentages represent the amount of variation in the data explained by the axis, calculated from the PCoA eigen values. Axes ranges represent the relative dissimilarity present between the samples. (TIF) [file pone.0213733.s006.tif]

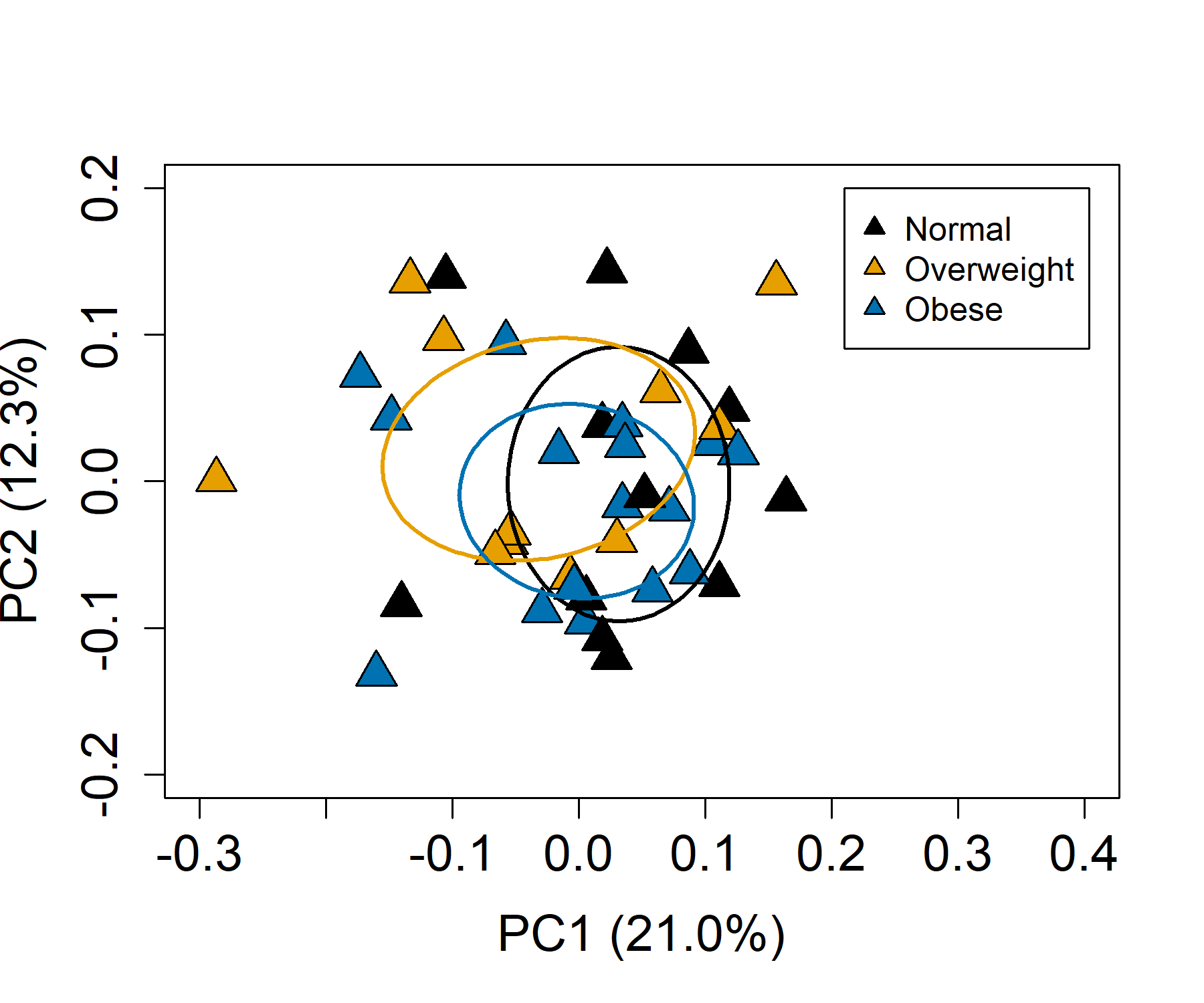

Supplement: S2 Fig — PCoA of the genus-level microbiota using Sorensen index. Axes percentages represent the amount of variation in the data explained by the axis, calculated from the PCoA eigen values. Axes ranges represent the relative dissimilarity present between the samples. (TIF) [file pone.0213733.s007.tif]

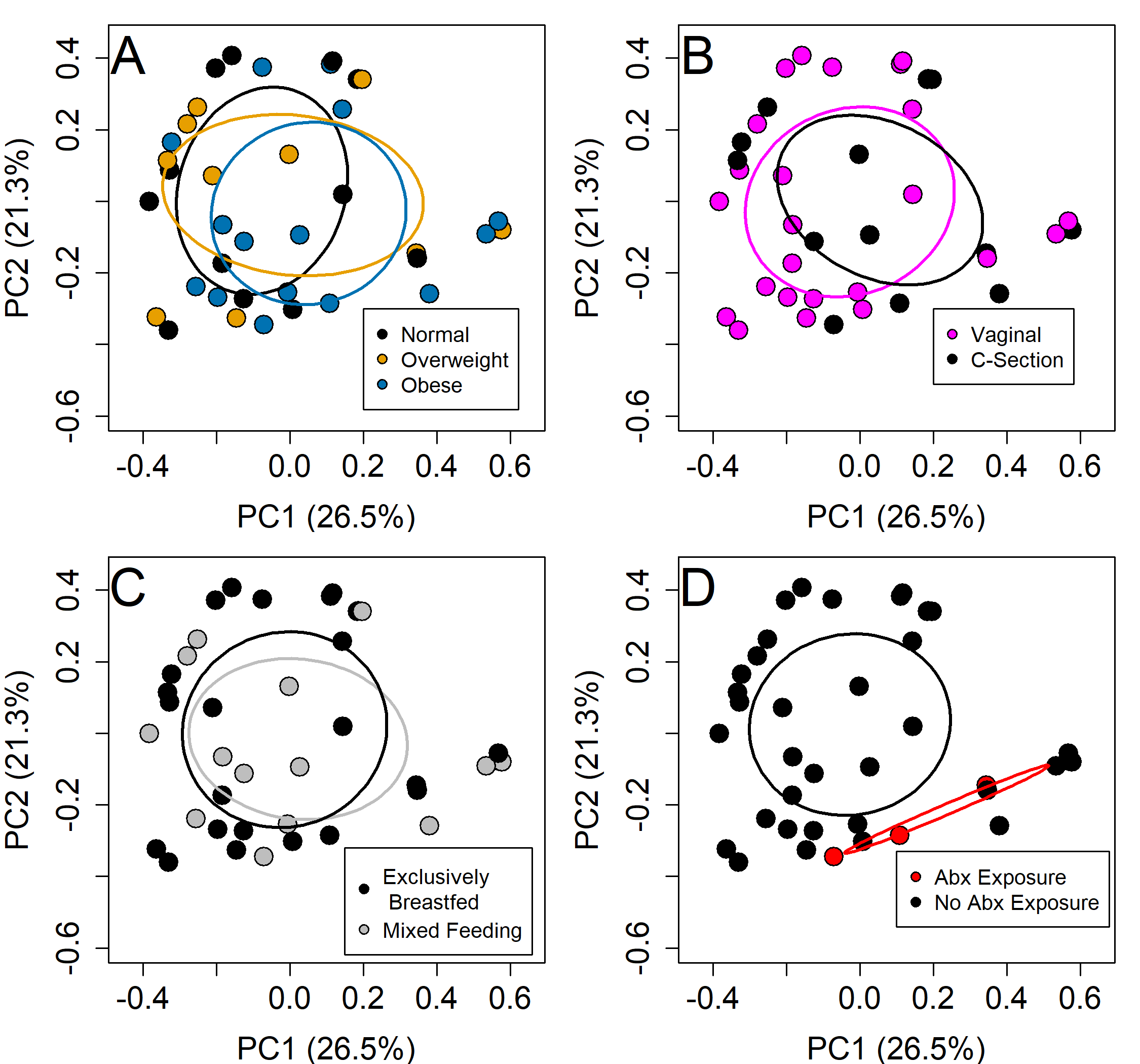

Supplement: S3 Fig — PCoA of the genus-level microbiota by Bray-Curtis dissimilarity comparing (A) maternal pre-pregnancy BMI category, (B) delivery mode, (C) breast milk in diet and (D) antibiotic exposure. Axes percentages represent the amount of variation in the data explained by the axis, calculated from the PCoA eigen values. Axes ranges represent the relative dissimilarity present between the samples. (TIF) [file pone.0213733.s008.tif]

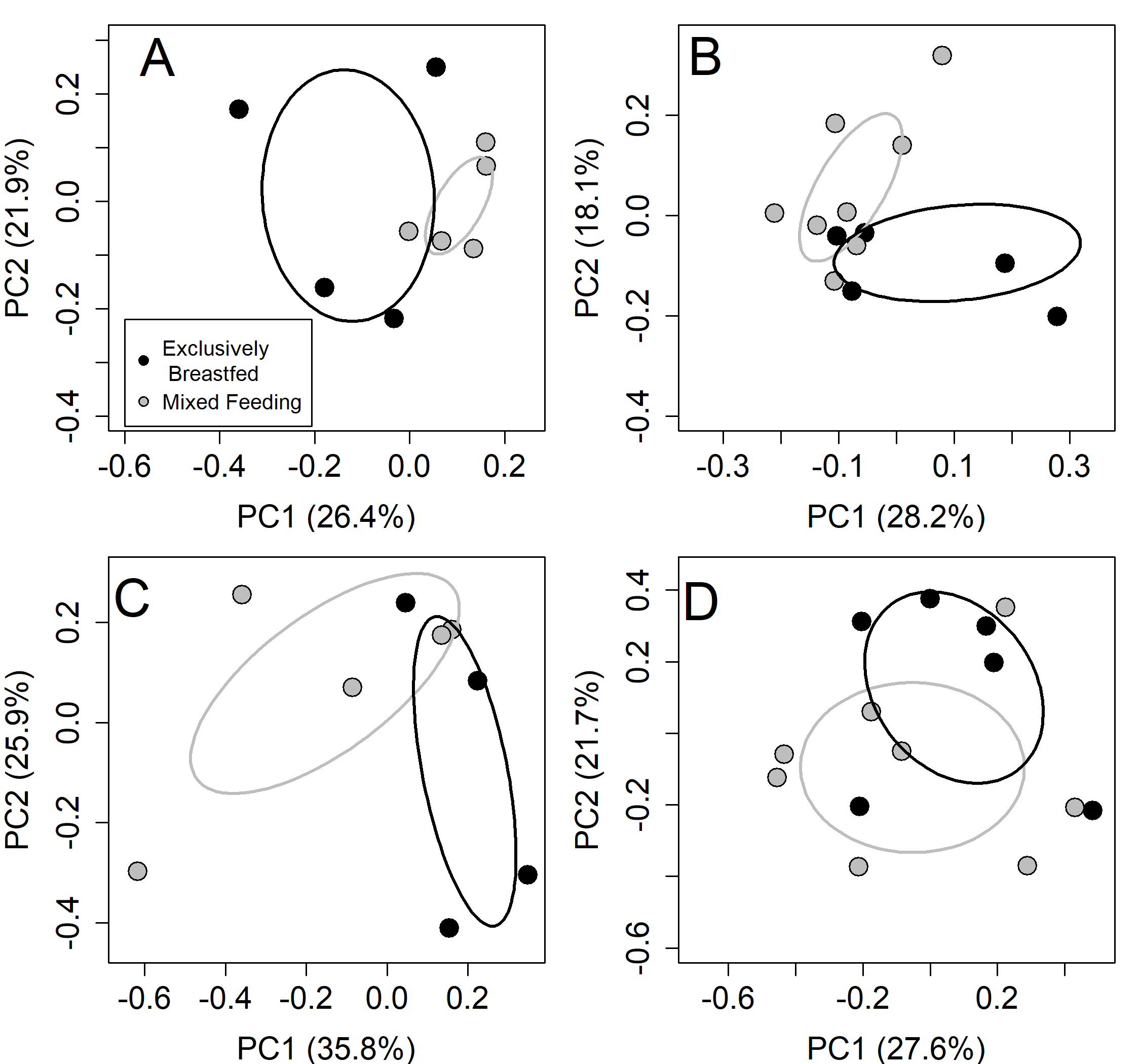

Supplement: S4 Fig — PCoA of the infant gut microbiota by breastfeeding status of the (A) overweight group using the Sorensen index at the genus level (B) obese group using the Sorensen index at the genus level (C) overweight group using Bray-Curtis dissimilarity at the phylum level (D) obese group using Bray-Curtis dissimilarity at the phylum level. Axes percentages represent the amount of variation in the data explained by the axis, calculated from the PCoA eigen values. Axes ranges represent the relative dissimilarity present between the samples. (TIF) [file pone.0213733.s009.tif]

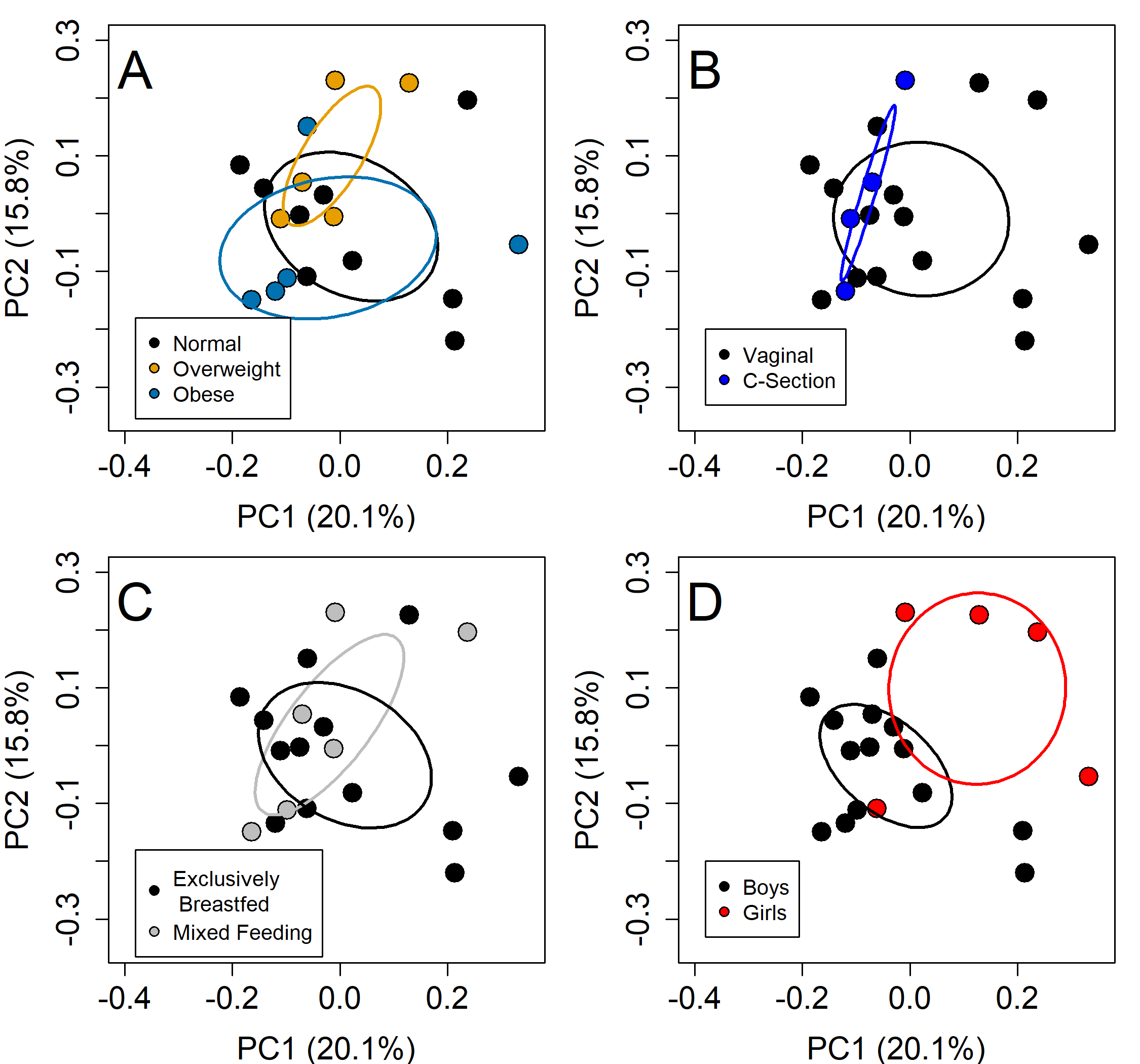

Supplement: S5 Fig — PCoA of the genus-level microbiota by Sorensen dissimilarity comparing (A) maternal pre-pregnancy BMI category, (B) delivery mode, (C) breastfeeding status and (D) sex. Axes percentages represent the amount of variation in the data explained by the axis, calculated from the PCoA eigen values. Axes ranges represent the relative dissimilarity present between the samples. (TIF) [file pone.0213733.s010.tif]

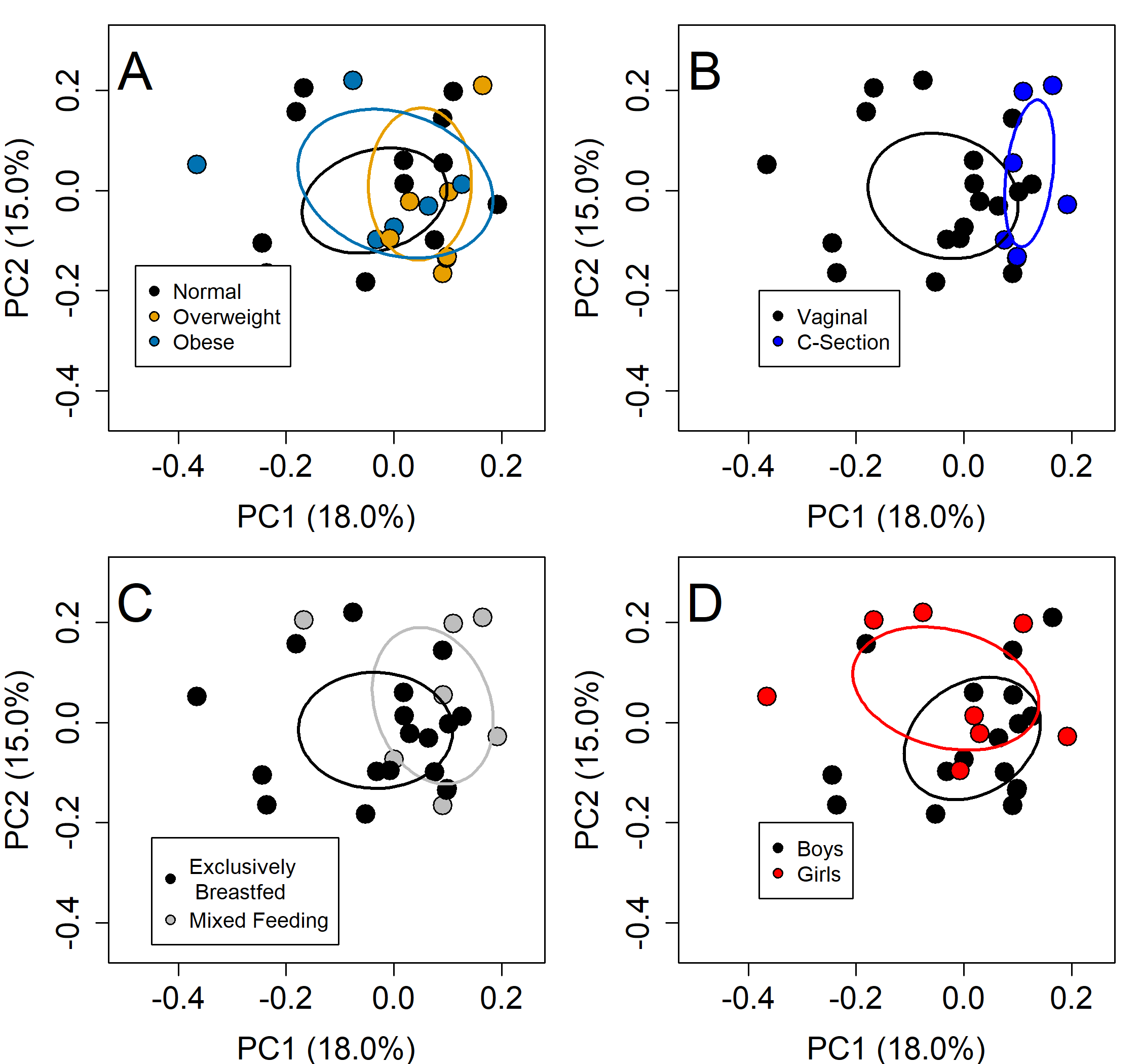

Supplement: S6 Fig — PCoA of the genus-level microbiota by Sorensen dissimilarity comparing (A) maternal pre-pregnancy BMI category, (B) delivery mode, (C) breastfeeding status and (D) sex. Axes percentages represent the amount of variation in the data explained by the axis, calculated from the PCoA eigen values. Axes ranges represent the relative dissimilarity present between the samples. (TIF) [file pone.0213733.s011.tif]
